# Underscreening and undertreatment? Periodontal service provision in very old Germans

**DOI:** 10.1007/s00784-020-03635-4

**Published:** 2020-10-23

**Authors:** Joachim Krois, Aleksander Krasowski, Jesus Gomez Rossi, Sebastian Paris, Adelheid Kuhlmey, Hendrik Meyer-Lückel, Falk Schwendicke

**Affiliations:** 1 Department of Oral Diagnostics, Digital Health and Health Services Research, Charité – Universitätsmedizin Berlin, corporate member of Freie Universität Berlin, Humboldt-Universität zu Berlin, and Berlin Institute of Health, Aßmannshauser Str. 4-6, 14197 Berlin, Germany; 2 Department of Restorative, Preventive and Pediatric Dentistry, zmk Bern, University of Bern, Bern, Switzerland; 3 Department of Operative and Preventive Dentistry, Charité – Universitätsmedizin Berlin, corporate member of Freie Universität Berlin, Humboldt-Universität zu Berlin, and Berlin Institute of Health, Berlin, Germany; 4 Institute of Medical Sociology and Rehabilitation Science, Charité – Universitätsmedizin, Berlin, corporate member of Freie Universität Berlin, Humboldt-Universität zu Berlin, and Berlin Institute of Health, Berlin, Germany

**Keywords:** Access, Geriatrics, Gerodontology, Health services research, Periodontology, Periodontal screening index

## Abstract

**Objectives:**

We aimed to assess periodontal services utilization in very old Germans.

**Methods:**

A comprehensive sample of very old (≥ 75 years), insured at a large Northeastern statutory insurer was followed over 6 years (2012–2017). We assessed periodontal service provision, entailing (1) periodontal screening index (PSI), (2) periodontal status/treatment planning, (3) periodontal therapy (scaling and root planning with or without access surgery), (4) postoperative reevaluation, and (5) any of these four services groups. Association of utilization with (1) sex, (2) age, (3) region, (4) social hardship status, (5) ICD-10 diagnoses, and (6) diagnoses-related groups was explored.

**Results:**

404.610 individuals were followed; 173,733 did not survive follow-up. The mean (SD) age was 81.9 (5.4) years. 29.4% (119,103 individuals) utilized any periodontal service, nearly all of them the PSI. Periodontal status/treatment planning, treatment provision, and reevaluation were provided to only a small fraction (1.54–1.57%, or 6224–6345) of individuals. The utilization of the PSI increased between 2012 and 2017; no such increase was observed for treatment-related services. Utilization decreased with age; those aged > 85 years received nearly no services at all. Decreases were more pronounced for treatment-related services. Utilization was lower in rural than urban areas, those with hardship status, and those severely ill (e.g., dementia, heart insufficiency). In multivariable analysis, a previous PSI measurement tripled the odds of receiving treatment-related services (OR: 3.2; 95% CI: 3.0-3.4).

**Conclusions:**

Periodontal services utilization was low. Screening for periodontal disease significantly increased therapy provision. Social, demographic, regional, and general health aspects were associated with utilization.

**Clinical significance:**

The utilization of periodontal services in the very old in Northeast Germany was low, and even screening was only performed in a minority of individuals. Policies to increase identification and management of periodontitis especially in the most vulnerable individuals are needed.

**Electronic supplementary material:**

The online version of this article (10.1007/s00784-020-03635-4) contains supplementary material, which is available to authorized users.

## Introduction

While general improvements in oral health in high income countries have clearly benefitted the majority of children and adults, indicated by fewer restored or missing teeth [1, 2], the same is not equally true for older adults. This group of individuals oftentimes retains a higher number of teeth into higher age, but concomitantly suffers from a higher number of coronal and root caries lesions as well as periodontally affected teeth. For Germany, for example, and the age group of 65–74 years old, a mean of 4.5 teeth showed periodontal pocketing (i.e., 4 mm or above) in 1997 (equaling 33.5 million teeth nationwide). This increased to 7.5 teeth (63.4 million) in 2014, and is projected to nearly double once more to 12.2 teeth (140.8 million) in 2030 [3].

Notably, the prevalence and extent of disease is not always reflected in the utilization of health services. A previous analysis found a mismatch between the amount of periodontal services provided in Germany and that expected to be provided based on epidemiological data (like the one above) [4]. Especially for the older individuals, it is likely that a range of further factors determine the utilization and provision of periodontal services. These may encompass age, general health, financial means, and place of living, all of which affect the accessibility of services. In a previous study and building on claims data, we found both prosthetic [5] and operative or surgical services utilization in older individuals to be affected by these aspects [6]. Moreover, we demonstrated that both the overall consumption of any service and the consumption of specific services are associated with such factors.

In the present study, we used claims data from one large insurer, mainly acting in the northeast of Germany, to evaluate the utilization of periodontal services in very old individuals (defined as those 75 years or older), and to associate this utilization with individuals’ age, general health, socioeconomic status, and place of living. While claims suffer from a range of limitations like selection, confounding, or misclassification bias, they allow to capture treatment patterns especially in those groups which are otherwise hard to assess (like the old, the sick, poor, and rural living ones), while concomitantly representing everyday care with limited risks of reporting bias and high generalizability for the investigated healthcare setting [7, 8].

## Methods

### Study design

For reasons of comparability, the employed methods and reporting of this study follows that of a previous publication on prosthetic treatment patterns in very old Germans [5]. The investigated cohort was evaluated based on claims data from a statutory (public) health insurance in Germany. Old individuals (75 years or older) from the AOK Nordost were followed over 6 years (2012 to 2017). The AOK Nordost is the Northeastern regional branch of a large national insurer, the Allgemeine Ortskrankenkasse (AOK), active mainly in the federal states of Berlin, Brandenburg, and Mecklenburg-Vorpommern. Notably, individuals may have moved away from these states during the observational studies; we excluded these for geographic analyses. The reporting of this study follows the RECORD statement [9].

### Setting

The AOK Nordost insures around 1.8 million individuals mainly in the German capital, Berlin, and two rural states, Brandenburg and Mecklenburg-Vorpommern, with only few larger cities (> 70,000 inhabitants). Data for this study were routinely collected and provided under ethical approval in a pseudonymized form using a data protection cleared platform via the scientific institute of the AOK Nordost, the GEWiNO.

### Participants and sample size

The target population comprised statutorily insured very old, aged 75 years or above, living in the Northeast of Germany, regardless if they utilized dental services or not. Hence, a comprehensive sample of very old, insured with the AOK Nordost in 2012, was drawn and followed over the 6 years observational period. No formal sample size estimation was performed given this being a comprehensive sample. Variable ascertainment was only possible via insurance base data and claims data. The database had been curated for plausibility by the GEWiNO.

### Variables

Our outcome was the utilization (in absolute numbers and in % of the population) of periodontal services. Within the statutory German insurance, dental services are provided on a fee-per-item basis using fee items catalogues of the statutory or private German insurance [10, 11]. The vast majority of patients are statutorily insured. For the statutory insurance, items are drawn from the fee item catalogue Bewertungsmaßstab (BEMA). For the present study, we only assessed a subset of items related to periodontal diagnostics and therapy, namely (1) periodontal screening index (BEMA 04), (2) periodontal status (six-point pocketing charting, which is standard for treatment planning within the statutory insurance) and treatment planning (BEMA 4), (3) periodontal therapy including closed and open scaling and root planing (P200–203), and (4) periodontal postoperative reevaluation (BEMA 111). We report on utilization of each of these services as well as any utilization (i.e., minimum 1 service). Notably, we did not assess the quantity of each consumed service (but only if it was consumed at all or not).

The utilization of services was assessed according to (1) sex (male/female); (2) age (in years) in each year of follow-up; (3) region, we used municipalities including the capital Berlin (with over 3.5 million inhabitants), medium-sized cities (70,000-200,000 inhabitants), and rural areas; (4) social hardship status (income < 1246 Euro/month per capita in 2019); (5) ICD-10 diagnoses, derived from outpatient diagnostic data; (6) inpatient hospital diagnoses and treatments, derived from German-Modified Diagnoses Related Groups (DRG). The DRGs classify diseases in groups of similar pathogenesis, characteristics, and treatment complexity. Only the 25 most frequently recorded ICD-10 and DRG codes were used.

### Data sources and access

As described before [5], data used for this study were provided by the GEWiNO using a data protection approved platform. Data were pseudonymized and included the described covariates and all BEMA items claimed per year among further variables. Comparability of data between different years and data consistency was given.

### Bias

Neither participants nor providers were aware that the collected claims data will be used for routine data analyses later on. Selection bias for the target population (very old individuals at AOK Nordost) was not possible within this study. Note, however, that the overall population of very old Germans differs and that our data likely submits to the mentioned biases of claims data, as discussed later on. No further measures against these biases could be taken.

### Statistical analyses

The statistical analysis was performed on the comprehensive sample (*n* = 404,610) of very olds insured at AOK Nordost in 2012. For the utilization of periodontal services, we considered an individual to have consumed a particular periodontal service (see above) if at least once during the observational period such a service was claimed. Descriptive statistics of age groups were computed based on the age distribution in 2012. An individual was assigned to having a social hardship if the individual was assigned to this status at least once during the period 2012 to 2017. For geographical analysis, we excluded all individuals that relocated from one of the federal states (Berlin, Brandenburg, and Mecklenburg-Vorpommern) to another federal state, thereby decreasing the sample size to 390,044. However, we did not correct for relocations within the three federal states during the observational period.

For each particular outpatient diagnosis (ICD-10 codes) and inpatient hospital diagnosis and treatment (DRGs), we summed up all claims, selected the 25 most frequent diagnoses each (in total 50) and computed for each of them the number of individuals that were assigned to having a diagnosis, respectively, treatment, during 2012 to 2017.

We applied logistic regression, a method to model a binary outcome variable as a linear combination of predictor variables. The response variable was the utilization of treatment-related services. As predictor variables we included age, gender, being deceased, the provision of the PSI, a social hardship status, federal state (we allowed the category “other” for relocated individuals) and the described outpatient and inpatient hospital diagnosis variables referring to the year 2012. All analyses, modeling and visualization were performed using Python (version 3.7, available at http://www.python.org) and auxiliary modules.

## Results

A total of 404,610 individuals were followed over up to 6 years (Table 1); 173,733 did not survive follow-up. The mean follow-up was 1689 days (standard deviation SD: 705). The mean (SD) age of the sample was 81.9 (5.4) years. The majority of individuals were female and younger than 85 years. Nearly half of them claimed hardship status once during the observational period. Of this cohort, 29.4% (119,103 individuals) utilized any periodontal service, nearly all of them the PSI. Periodontal status/treatment planning, treatment provision, and reevaluation were provided to only a small fraction (1.54–1.57%, or 6224–6345 individuals). Males received all services slightly more often than females, younger individuals more often than older ones, those without hardships status more often than those with hardship status, and those in Berlin more often than those in more rural Brandenburg or Mecklenburg-Vorpommern.

**Table 1:** Sample characteristics (N; %) from Northeast Germany. Total, male, and female population aged 75 years or older, in 5-year age bands and according to federal state

Covariate	Group	*N*	Any	PSI	Status/planning	Treatm.	Reeval.
All		404610 (100.0)	119103 (29.4)	118537 (29.3)	6333 (1.6)	6345 (1.6)	6224 (1.5)
Gender	Male	134909 (33.3)	43187 (32.0)	42968 (31.8)	2394 (1.8)	2396 (1.8)	2350 (1.7)
	Female	269702 (66.7)	75916 (28.1)	75569 (28.0)	3939 (1.5)	3949 (1.5)	3874 (1.4)
Age group	75–79	162368 (22.7)	50457 (31.1)	49951 (30.8)	3307 (2.0)	3310 (2.0)	3246 (2.0)
	80–84	266956 (37.4)	70133 (26.3)	69669 (26.1)	2619 (1.0)	2632 (1.0)	2584 (1.0)
	85–89	174673 (24.5)	32667 (18.7)	32540 (18.6)	673 (0.4)	676 (0.4)	663 (0.4)
	90–94	82597 (11.6)	9336 (11.3)	9325 (11.3)	82 (0.1)	81 (0.1)	79 (0.1)
	95–99	22641 (3.2)	1359 (6.0)	1358 (6.0)	5 (0.0)	5 (0.0)	5 (0.0)
	100–104	4214 (0.6)	125 (3.0)	124 (2.9)	1 (0.0)	1 (0.0)	1 (0.0)
	105–109	348 (0.0)	9 (2.6)	9 (2.6)	0 (0.0)	0 (0.0)	0 (0.0)
Social hardship status	No	210292 (52.0)	63218 (30.1)	62900 (29.9)	3680 (1.7)	3687 (1.8)	3617 (1.7)
	Yes	194318 (48.0)	55885 (28.8)	55637 (28.6)	2653 (1.4)	2658 (1.4)	2607 (1.3)
Federal state	Berlin	122454 (30.3)	41798 (34.1)	41594 (34.0)	2163 (1.8)	2167 (1.8)	2138 (1.7)
	Brandenburg	153164 (37.9)	43282 (28.3)	43125 (28.2)	2234 (1.5)	2234 (1.5)	2188 (1.4)
	Mecklenburg	107665 (26.6)	27880 (25.9)	27702 (25.7)	1654 (1.5)	1662 (1.5)	1623 (1.5)
	Others*	21327 (5.3)	6143 (28.8)	6116 (28.7)	282 (1.3)	282 (1.3)	275 (1.3)

*“Others” indicates individuals who were insured at AOK Nordost, but did not live in the three federal states of interest. These were excluded from geographic analyses (Fig. 2)

**Fig. 1:**
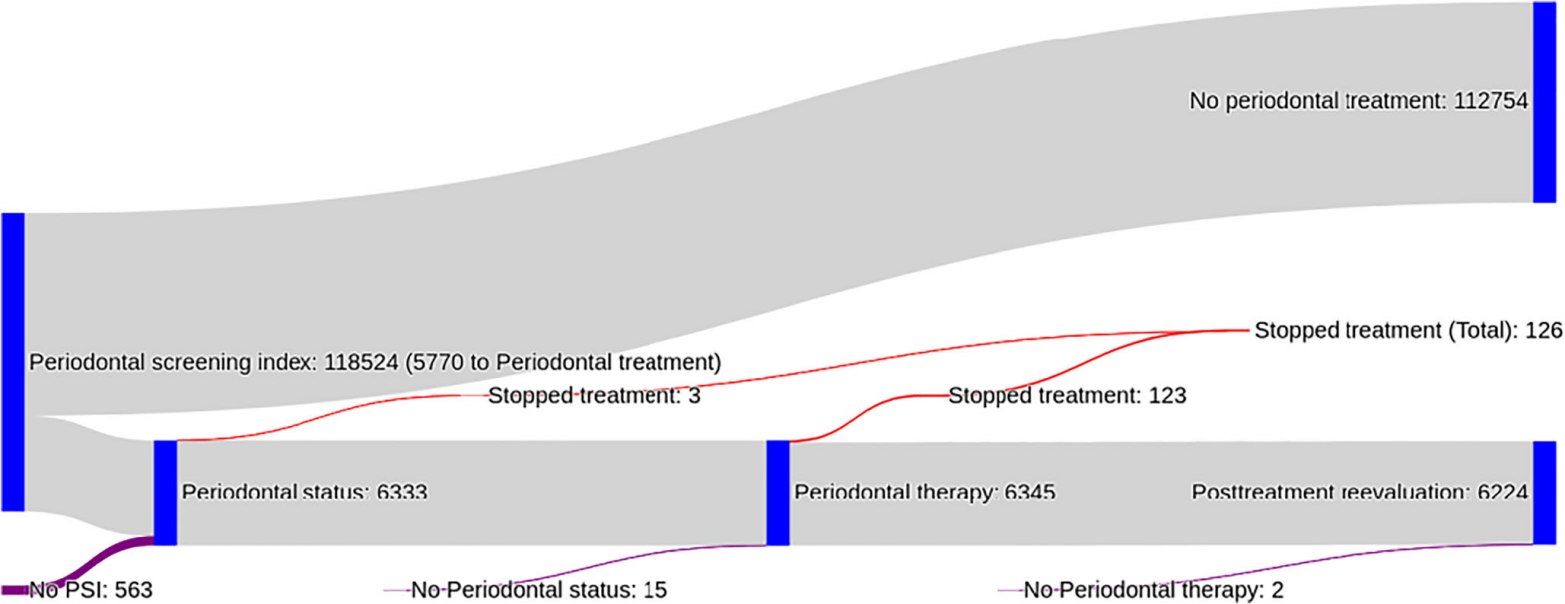
Flow of very old individuals from Northeast Germany through the different stages of periodontal therapy. From those who were screened, a minority were evaluated for periodontal status and treatment planning. Those who received this service, though, nearly all also received therapy and reevaluation

Utilization was determined largely by measurement of the PSI, 99.5% (118,537) of all patients receiving any periodontal services had been provided with the PSI. Only a fraction of these eventually received treatment-related care, as described. For those who did receive treatment-related care, a comprehensive set of items including status/planning over treatment (near exclusively nonsurgical treatment) and postoperative reevaluation was claimed (Fig. 1).

**Fig. 2:**
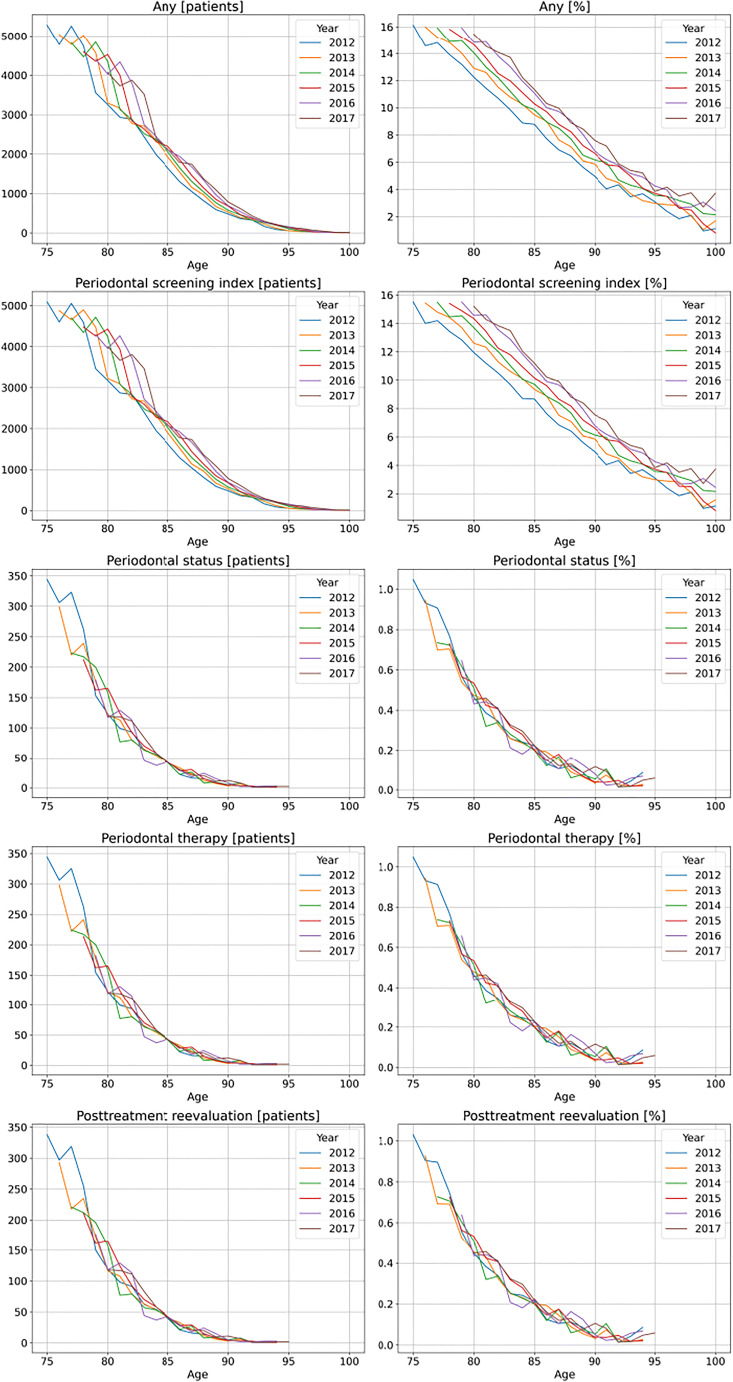
Utilization (*N*, and in %) of periodontal services by the very old in Northeast Germany. Any utilization and specific periodontal services utilization are shown. Individuals available in 2012 of all ages from 75 years upwards (blue line) were followed over 6 years until 2017 (black line), i.e., the 75 years in 2012 are the 76 years in 2013 etc. (which is why the lines start further to the right with longer follow-up).

When assessing utilization over time and in different age groups (Fig. 2), a number of trends were identified: The utilization of the PSI increased between 2012 and 2017, e.g., 8.4% of all 85-year-olds were provided with PSI measurements in 2012, while the same age group showed a 11.0% utilization in 2017 (i.e., a relative increase by 30%). Over their remaining lifetime, individuals reduced the utilization of the PSI near linearly. As the PSI was the main driver of the overall utilization of periodontal services (see above), a similar pattern was observed when assessing the utilization of any periodontal services (Fig. 2). For all other services, a near identical behavior was noted: No increase over time was identified, and utilization decreased in a more exponential fashion when individuals got older, i.e. it halved between age 75 and 80 years, halved once more between 80 and 85 years old and was then near zero for the remaining life years.

**Fig. 3:**
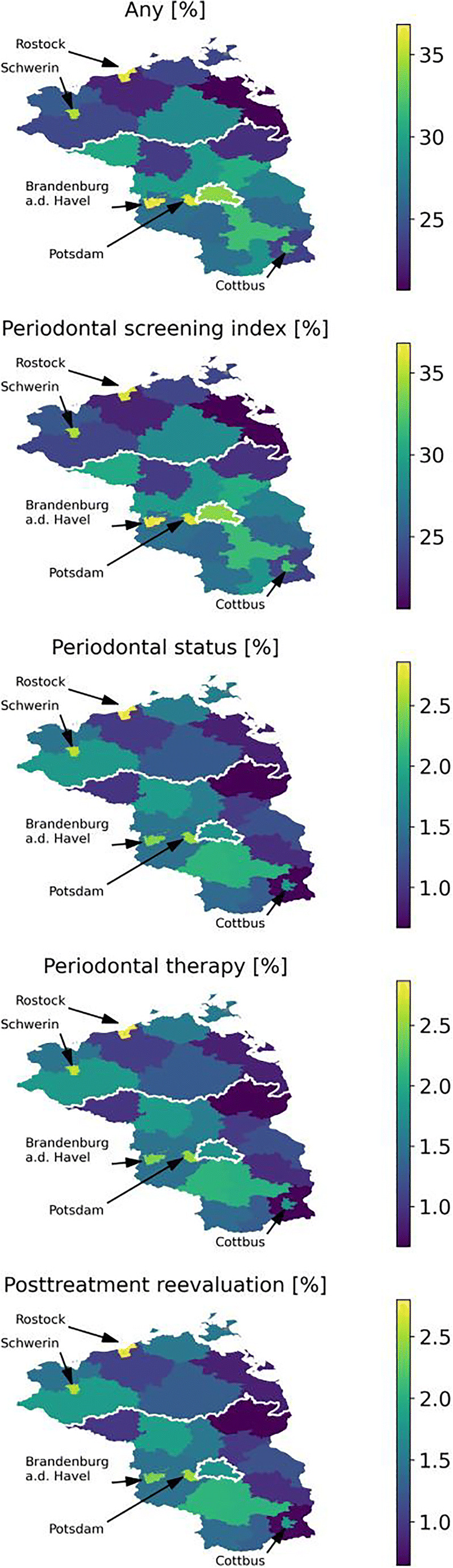
Regionally specific utilization of periodontal services, stratified in services blocks, in Northeast Germany. Relative (in %) any utilization and specific periodontal services utilization is shown. Larger cities with an increased or decreased utilization compared to the surrounding municipalities are further highlighted by arrows

Large geographical disparities were identified (Fig. 3). For example, the PSI utilization exceeded 35% in some cities, but was below 20% in some rural municipalities in the Eastern parts of Mecklenburg-Vorpommern. Generally, this state showed lower utilization than Berlin and Brandenburg. For all other services except the PSI, utilization was higher in cities and in Western than Eastern municipalities.

Utilization of periodontal services was assessed according to ICD-10 codes (Table 2). Utilization of any services and PSI was higher for the majority of codes, especially eye conditions (e.g., presbyopia, cataract, astigmatism), but significantly lower for heart insufficiency, urinary incontinence, and dementia. A similar pattern was found for treatment-related services, while notably, the relative differences were aggravated, i.e., treatment-related services utilization decreased more pronounced in the very sick ones than utilization of the PSI.

**Table 2:** Utilization of periodontal services according to International Disease Classification (ICD-10, German Modification) codes by the very old in Northeast Germany. Total population with specific ICD-10 code (*N*) and numbers (%) of those using any periodontal services, periodontal screening index (PSI), periodontal treatment, or periodontal reevaluation is shown

ICD-10-GM	Outpatient diagnoses code	Description	*N*	Any	PSI	Status/planning	Treatm.	Reeval.
-	Total	-	404610 (100.0)	119103 (29.4)	118537 (29.3)	6333 (1.6)	6345 (1.6)	6224 (1.5)
-	UUU	Special cases, without diagnostic certainty (e.g. passing on findings or replying to health insurance enquiries or order-related services)	374109 (92.5)	117363 (31.4)	116805 (31.2)	6281 (1.7)	6293 (1.7)	6172 (1.6)
Diseases of the circulatory system	I10.90	Essential hypertension, not further described	342446 (84.6)	105704 (30.9)	105201 (30.7)	5647 (1.6)	5656 (1.7)	5549 (1.6)
Factors influencing health status and contact with health services	Z25.1 *	The need for vaccination against influenza	258991 (64.0)	86188 (33.3)	85809 (33.1)	4595 (1.8)	4602 (1.8)	4515 (1.7)
Endocrine, nutritional and metabolic diseases	E11.90	Diabetes mellitus, type 2 without complications - Not designated as derailed	176727 (43.7)	51118 (28.9)	50885 (28.8)	2648 (1.5)	2654 (1.5)	2599 (1.5)
Diseases of the eye and adnex	H52.4	Presbyopia	169474 (41.9)	65977 (38.9)	65649 (38.7)	3768 (2.2)	3778 (2.2)	3710 (2.2)
Diseases of the eye and adnex	H52.2	Astigmatism	161643 (40.0)	63266 (39.1)	62945 (38.9)	3655 (2.3)	3662 (2.3)	3596 (2.2)
Diseases of the circulatory system	I25.9	Chronic ischaemic heart disease, not further specified	160456 (39.7)	48338 (30.1)	48115 (30.0)	2382 (1.5)	2380 (1.5)	2333 (1.5)
Diseases of the musculoskeletal system and connective tissue	M17.9	Gonarthrosis, not further described	141200 (34.9)	49664 (35.2)	49442 (35.0)	2654 (1.9)	2661 (1.9)	2610 (1.8)
Diseases of the circulatory system	I10.00	Benign essential hypertension - no indication of a hypertensive crisis	140812 (34.8)	49499 (35.2)	49271 (35.0)	2665 (1.9)	2669 (1.9)	2612 (1.9)
Diseases of the eye and adnex	H52.0	Accommodation disorders and refraction errors	139335 (34.4)	55276 (39.7)	54984 (39.5)	3255 (2.3)	3261 (2.3)	3201 (2.3)
Factors influencing health status and contact with health services	Z96.1 *	Presence of an intraocular lens implant	137658 (34.0)	51537 (37.4)	51305 (37.3)	2751 (2.0)	2758 (2.0)	2714 (2.0)
Diseases of the eye and adnex	H26.9	Cataract, not further specified	134910 (33.3)	50248 (37.2)	50017 (37.1)	2873 (2.1)	2880 (2.1)	2827 (2.1)
Symptoms, signs and abnormal clinical and laboratory findings, not elsewhere classified	R32 **	Unknown urinary incontinence	129305 (32.0)	32240 (24.9)	32116 (24.8)	1264 (1.0)	1267 (1.0)	1236 (1.0)
Mental and behavioural disorders	F03	Undescribed dementia	127647 (31.5)	28077 (22.0)	27974 (21.9)	960 (0.8)	963 (0.8)	939 (0.7)
Symptoms, signs and abnormal clinical and laboratory findings, not elsewhere classified	R52.2 **	Other chronic pain	125881 (31.1)	43976 (34.9)	43797 (34.8)	2189 (1.7)	2192 (1.7)	2145 (1.7)
Diseases of the circulatory system	I50.9	Heart failure, not further specified	118705 (29.3)	31226 (26.3)	31098 (26.2)	1223 (1.0)	1224 (1.0)	1201 (1.0)
Endocrine, nutritional and metabolic diseases	E78.5	Hyperlipidemia, not further described	114440 (28.3)	39669 (34.7)	39484 (34.5)	2297 (2.0)	2297 (2.0)	2247 (2.0)
Diseases of the musculoskeletal system/connective tissue	M16.9	Coxarthrosis, not further described	112428 (27.8)	39417 (35.1)	39262 (34.9)	1981 (1.8)	1983 (1.8)	1945 (1.7)
Endocrine, nutritional and metabolic diseases	E78.0	Pure hypercholesterolemia	101100 (25.0)	37750 (37.3)	37552 (37.1)	2242 (2.2)	2244 (2.2)	2200 (2.2)
Diseases of the circulatory system	I70.9	Generalized and unspecified atherosclerosis	95316 (23.6)	31705 (33.3)	31581 (33.1)	1624 (1.7)	1626 (1.7)	1594 (1.7)
Diseases of the musculoskeletal system and connective tissue	M81.99	Osteoporosis, not further described - not further described Localization	95150 (23.5)	31921 (33.5)	31769 (33.4)	1607 (1.7)	1612 (1.7)	1588 (1.7)
Factors influencing health status and contact with health services	Z92.1 *	Long-term therapy (present) with anticoagulants in the patient's own medical history	89815 (22.2)	31126 (34.7)	31016 (34.5)	1500 (1.7)	1501 (1.7)	1470 (1.6)
Diseases of the circulatory system	I83.9	Varices of the lower extremities without ulceration or inflammation	88616 (21.9)	31935 (36.0)	31797 (35.9)	1737 (2.0)	1740 (2.0)	1709 (1.9)
Endocrine, nutritional and metabolic diseases	E79.0	Hyperuricemia without signs of inflammatory arthritis or tophic gout	77737 (19.2)	25038 (32.2)	24926 (32.1)	1237 (1.6)	1238 (1.6)	1215 (1.6)
Diseases of the genitourinary system	N40	Prostatic hyperplasia	74931 (18.5)	27904 (37.2)	27773 (37.1)	1541 (2.1)	1543 (2.1)	1514 (2.0)

We further assessed the utilization of periodontal service stratified according to different DRGs (Table 3). A complex utilization pattern was observed: Any and PSI utilization was decreased to some degree (at max. by ca. 30%) for patients with severe illnesses, e.g., severe heart insufficiency and esophagitis/ulcerations or infections, while it was similar increased (by nearly 50%) for patients with nonsevere bronchitis, nonsevere hypertensions, or syncope. The utilization of treatment-related services was decreased in a wider number of patients, e.g., those with severe heart insufficiency, esophagitis/ulcerations, infections, metabolic diseases, injuries, joint replacements, chronic obstructive pulmonary disease and geriatric rehabilitation, and these decreases were relatively more pronounced (e.g., more than halved for many diagnoses). Utilization of treatment-related services was increased for patients with bronchitis.

**Table 3:** Utilization of periodontal services by the very old in Northeast Germany according to German Modified Diagnosis related groups (DRG). Total population with specific DRG (*N*) and numbers (%) of those using any periodontal services, periodontal screening index (PSI), periodontal treatment, or periodontal reevaluation is shown

GM-DRG	Description	N	Any	PSI	Status/planning	Treatm.	Reeval.
-	Total population	404610 (100.0)	119103 (29.44)	118537 (29.3)	6333 (1.57)	6345 (1.57)	6224 (1.54)
F62B	Cardiac insufficiency and shock with extremely serious complications or comorbidity, with dialysis or complicated diagnosis or with certain high-level treatment or without complicated constellation, without specific high-level treatment, more than 1 day of occupancy in certain acute renal failure with extremely severe complications or comorbidity	40295 (10.0)	8765 (21.8)	8731 (21.7)	261 (0.6)	261 (0.6)	253 (0.6)
G67C	Esophagitis, gastroenteritis, gastrointestinal hemorrhage, ulcer disease and various diseases of the digestive organs without certain or other complicating factors, without extremely severe complications or comorbidity	21650 (5.4)	6444 (29.8)	6425 (29.7)	249 (1.2)	250 (1.2)	245 (1.1)
I41Z	Geriatric early rehabilitative complex treatment for diseases and disorders of the musculoskeletal system and connective tissue	21131 (5.2)	6530 (30.9)	6507 (30.8)	203 (1.0)	204 (1.0)	196 (0.9)
K62B	Various metabolic diseases in paraplegia / tetraplegia or with complicated diagnosis or endoscopic insertion of a gastric balloon or age < 16 years, one occupancy day or without extremely severe complications or comorbidity or without certain costly / highly complex treatment	19998 (4.9)	5699 (28.5)	5685 (28.4)	228 (1.1)	229 (1.1)	223 (1.1)
G67B	Esophagitis, gastroenteritis, gastrointestinal bleeding, ulcer disease and various diseases of the digestive organs with other complicating factors or with extremely severe complications or comorbidity	19637 (4.9)	4395 (22.4)	4380 (22.3)	158 (0.8)	158 (0.8)	149 (0.8)
F71B	Non-severe cardiac arrhythmias and conduction disturbances without extremely severe complications or comorbidity or occupancy day, without catheter-assisted electrophysiological examination of the heart, without specific high-level treatment	16666 (4.1)	6220 (37.3)	6196 (37.2)	310 (1.9)	310 (1.9)	305 (1.8)
F67D	Hypertension without complicated diagnosis, without extremely severe or severe complications or comorbidity, without certain moderately complex / complicated treatment, age > 17 years	15300 (3.8)	5370 (35.1)	5346 (34.9)	264 (1.7)	265 (1.7)	260 (1.7)
E77I	Infections and inflammation of the respiratory system without complex diagnosis, without extremely severe complication or comorbidity or a complication or comorbidity, age > 0 years, except for para / quadriplegia, without complex treatment in multidrug-resistant pathogens	15143 (3.7)	3522 (23.3)	3507 (23.2)	114 (0.8)	115 (0.8)	113 (0.7)
F48Z	Geriatric early rehabilitative complex treatment for diseases and disorders of the circulatory system	14512 (3.6)	3792 (26.1)	3782 (26.1)	99 (0.7)	99 (0.7)	93 (0.6)
L63F	Infections of the urinary organs without extremely severe complications or comorbidity, without certain moderately costly / elaborate / highly costly treatment, without complex treatment multi-resistant pathogens (MRE), without certain serious infections, age > 5 and < 18 years, without severe complications or comorbidity or age > 17 and < 90 years	13704 (3.4)	3341 (24.4)	3329 (24.3)	115 (0.8)	115 (0.8)	111 (0.8)
I68D	Non-surgically treated diseases and injuries of the spinal column, more than one occupancy day or other femoral fracture, without sacrum fracture, without certain moderately elaborate, elaborate or highly elaborate treatment	12645 (3.1)	3629 (28.7)	3614 (28.6)	134 (1.1)	135 (1.1)	134 (1.1)
F73Z	Syncope and collapse	12352 (3.1)	4642 (37.6)	4624 (37.4)	228 (1.8)	228 (1.8)	224 (1.8)
B80Z	Other head injuries	11645 (2.9)	3146 (27.0)	3137 (26.9)	112 (1.0)	112 (1.0)	110 (0.9)
L60D	Renal insufficiency, more than one occupancy day, without dialysis, without extremely severe complications or comorbidity, age > 17 years or without severe complications or comorbidity, without complex intensive care treatment >196 / 184 / - expense points	10975 (2.7)	4137 (37.7)	4116 (37.5)	209 (1.9)	210 (1.9)	208 (1.9)
I47B	Revision or replacement of the hip joint without certain complicated factors, with complex diagnosis of the pelvis/thigh, with certain endoprosthetic or joint plastic surgery of the hip joint, with implantation or replacement of a radius head prosthesis.	10969 (2.7)	2539 (23.1)	2526 (23.0)	81 (0.7)	81 (0.7)	78 (0.7)
J65Z	Injury of the skin, subcutis and mamma	10541 (2.6)	2749 (26.1)	2741 (26.0)	89 (0.8)	89 (0.8)	88 (0.8)
E65C	Chronic obstructive pulmonary disease without extremely severe complication or comorbidity, without complicated diagnosis, without FEV1 < 35% or a complication or comorbidity, age > 1 year, without specific moderately complex / expensive treatment	10058 (2.5)	2412 (24.0)	2406 (23.9)	74 (0.7)	74 (0.7)	74 (0.7)
C08B	Extracapsular extraction of the lens (ECCE) without congenital malformation of the lens or certain interventions on the lens	9831 (2.4)	3755 (38.2)	3744 (38.1)	144 (1.5)	144 (1.5)	135 (1.4)
A90A	Partial stationary geriatric complex treatment	9830 (2.4)	2421 (24.6)	2412 (24.5)	91 (0.9)	91 (0.9)	86 (0.9)
E69B	Bronchitis and bronchial asthma, more than 1 day of treatment. Age > 55 years or with extremely severe or severe complication or comorbidity, age > 0 years or 1 day of treatment or without extremely severe or severe complication or comorbidity, age < 1 year or flexible bronchoscopy, age < 16 years or determined moderate treatment, with RS virus -Infection.	9777 (2.4)	4530 (46.3)	4498 (46.0)	310 (3.2)	312 (3.2)	308 (3.2)
F49G	Invasive cardiological diagnosis except in acute myocardial infarction, without extremely severe complication or comorbidity, age > 17 years, without cardiac mapping, without severe complication or comorbidity at day of treatment>1, without complex diagnosis, without specific intervention	9574 (2.4)	3430 (35.8)	3421 (35.7)	143 (1.5)	143 (1.5)	143 (1.5)
I34Z	Geriatric early rehabilitative complex treatment with specific operating room procedure for diseases and disorders of the musculoskeletal system and connective tissue	9419 (2.3)	2753 (29.2)	2750 (29.2)	93 (1.0)	93 (1.0)	92 (1.0)
F62D	Cardiac insufficiency and shock without extremely serious complications or comorbidity or without dialysis, without complicated diagnosis, without complicated constellation, without specific high-level treatment, 1 day of occupancy	8615 (2.1)	2745 (31.9)	2735 (31.7)	106 (1.2)	106 (1.2)	104 (1.2)
B70B	Apoplexy with neurological complex treatment of acute stroke, more than 72 hours, without complicated diagnosis or with complex cerebrovascular vasospasm or intensive care complex treatment	8499 (2.1)	2386 (28.1)	2371 (27.9)	100 (1.2)	99 (1.2)	96 (1.1)
L64A	Other urinary organ diseases with extremely severe or severe complications or comorbidity or certain diagnosis, more than one occupancy day or urethra-cystoscopy, congenital malformation or age < 3 years	5669 (1.4)	1611 (28.4)	1602 (28.3)	77 (1.4)	77 (1.4)	75 (1.3)

In multivariable analysis (Table 4), individuals with a previous PSI measurement had a more than three times increased odds of receiving further treatment-related services (OR: 3.20; 95% CI: 3.03-3.38) Vice versa, utilization of treatment-related services was lower for individuals living outside of Berlin, e.g. Mecklenburg-Vorpommern: (0.89; 0.83-0.96). It was also decreased in individuals with social hardship status (0.80; 0.76-0.85), older individuals (0.86; 0.86-0.87 per year of age), and those who died during the observational period (0.37; 0.34-0.41). A range of comorbidities according to ICD-10 and DGRs also showed significant associations, mostly these association were of limited magnitude, though (Table 4, Table S1). Pseudo-*R*^2^ indicated that the model generally had limited explanatory power (*R*^2^ = 0.12).

**Table 4:** Multivariable analysis of factors associated with utilization of periodontal treatments

*Covariate*	*OR*	*2.5%*	*97.5%*	*p value*
Gender [male]	1.023	0.958	1.091	0.499
Social hardship status [yes]	*0.801*	*0.758*	*0.846*	*< 0.001*
PSI previously measured [yes]	*3.199*	*3.032*	*3.376*	*< 0.001*
Deceased [yes]	*0.372*	*0.340*	*0.406*	*< 0.001*
Federal state [Brandenburg]	*0.874*	*0.820*	*0.931*	*< 0.001*
Federal State [Mecklenburg-Vorpommern]	*0.894*	*0.833*	*0.960*	*0.002*
Federal State [Other]	0.993	0.874	1.129	0.919
Age [year]	*0.862*	*0.855*	*0.869*	*< 0.001*

We here show abbreviated results, excluding the association with general medical conditions (the full results can be found in the appendix). Significant associations are shown in italics

## Discussion

In the present study, we assessed periodontal services utilization by very old individuals in the northeast of Germany. Given the expected morbidity growth in this age group, mainly via morbidity compression, and given that this age group itself is growing, such analysis seems warranted, even more as so the utilization in this group is affected by multiple factors, like age, gender, general health, social status, and place of living [12, 13]. Understanding service utilization in specific populations or groups may allow developing targeted programs for increasing access in those with high needs and current under-utilization, thereby improving health services’ efficacy, efficiency and equitability [14]. We confirmed our hypothesis that social, demographic, regional, and general health aspects were associated with the utilization of periodontal services in very old Germans; these findings are in line with previous ones on other dental services, as discussed below [5]. More relevant for this specific investigation, though, was that in this presumably high-need population, only a small fraction of individuals were screened for periodontitis (via the PSI) and of those who were screened, an even smaller fraction eventually received treatment. It is possible that in this specific age group, dentists “adjust” the conventional thresholds for treatment initiation, i.e., focus on severe cases, and that patients similarly adopt their priorities for dental therapy, as discussed below.

More generally, a low utilization of periodontal services has been found on a national level and across age groups before [15]; the national average annual utilization (largely determined by the PSI, which is similar to our study) is estimated at around 23%; this number shrinks to 1.5% if only assessing treatment-related services. In our population, a nearly identical treatment-related utilization (1.6%) and an only minimally higher overall utilization (29.2%) was found over 6 years; i.e., the annual utilization is likely much smaller in the elderly in the northeast than in the national average. Note that this is also extremely low when comparing it with the overall utilization of dental services in this population, which we estimated at 73% for this cohort over the observational period [6]. Given that the PSI is allowed to be provided on a two-yearly basis for dentate patients, whose share in the very old is > 65% [16], and given that > 80% of the dentate very olds are (at least mild) periodontitis patients [3], it is clear that screening and, much more so, treatment are by far underutilized in the population of interest. The latter might be grounded in the high hurdles installed prior to periodontal therapy, which oftentimes require active cooperation of patients and, in many cases, out-of-pocket expenses (e.g., for professional tooth cleaning). The relevance of such hurdles (and potentially further factors, which the present study cannot consider) are confirmed when comparing utilization in a similar group of individuals, namely patients with dementia in Sweden. Based on health register data from a similarly very old (in mean 80 years), rather female and very sick population found an annual utilization of periodontal services of over 50%, i.e., much higher [17].

A number of aspects require deeper discussion. First, the conceptual chain between screening, status/planning, treatment, and follow-up seems to be broken after screening, but intact in the subsequent steps: Nearly all patients who underwent a full charting and treatment planning eventually received treatment and follow-up reevaluation. Notably, treatment was with only very few exceptions (126 cases for the whole population) provided conservatively, i.e., possible surgical follow-up therapies after the initial root scaling and planing was not provided often. This was our reason for not showing these data separately, while the underlying barriers to provide or receive surgical care are beyond this analysis.

Second, the utilization of the PSI has been significantly increasing during the follow-up period. As screening is the first step towards (and the strongest predictor for) treatment, this trend is positive and aligns with political goals of German dental health policy-makers: The German Dental Association, for instance, is currently preparing a periodontitis awareness campaign (www.bzaek.de). Based on our numbers, a more dramatic impact on utilization, though, can be expected when successfully reducing the screening-treatment gap.

Third, we identified an age-specific decline in utilization; this is in line with previous analyses on this cohort but also national estimates [5, 15]. Notably, utilization of the PSI decreased less pronounced (in a more linear fashion) than utilization of treatment-related services (which decreased exponentially); treatment was nearly never provided for individuals aged 85 years or older. This might indicate that either the effort to undergo treatment was inacceptable to patients or that priorities realigned from long-term tooth retention to maintenance of a pain-free dentition (or functioning denture). From a health services perspective, it can be questioned, then, why screening is performed at all. Given our previous findings that outreach care is increasing with age, and assuming periodontal care being one of the therapies which can be provided during outreach visits [6], it is notable that periodontal therapy does not seem to be provided during outreach visits; these visits seem to be rather focused on minimal care (including alleviation of pain or refitting/rebasing/repairing of removable dentures) than on regular tooth-retaining care.

Fourth, age is associated with an increasing prevalence of diseases or hospitalization [18]. In line with our previous analysis on operative, surgical, and prosthetic services, individuals with severe health conditions (e.g., severe gastrointestinal ulcerations, dementia, severe heart insufficiency) showed decreased utilization, while those with nonsevere condition, most prominently those of the eyes, showed increased utilization. We assume two different type of elderly to be underlying this finding; the very sick who are physically unable to attend the dentist and prioritize their general care, and the rather fit ones who are willing to receive eye care but also dental therapy to maintain a high quality of life. Identifying different groups early one may assist in planning appropriate care for each older individual. From the specific focus of this study, it is again unfortunate that the sick ones, who are likely to require specific attention on periodontitis diagnosis and treatment, are less likely to receive both.

Fifth, we assessed the impact of social hardship status on utilization. Hardship status is a proxy for low income but does otherwise not directly impact on accessibility of dental care in Germany except for prosthetic care (where additional costs are decreased when having this status; this was found with an increased utilization in the investigated cohort) [5]. In line with previous research, hardship status was found to be associated with decreased utilization, possibly acting as marker of not only low income but also low health literacy and unfavorable health behaviors. As those with low social status are also likely to show the poorest oral and general health [19], we again confirm a highly inequitable utilization of periodontal services.

Last, we found pronounced differences in utilization according to place of living [14]. Utilization was highest in Berlin and lowest in Mecklenburg-Vorpommern, and generally higher in cities than rural municipalities. Such disparity has been assumed to be grounded in workforce shortages or limited geographic accessibility, for example aggravated by thin public transport networks, in rural compared with urban areas. Moreover, urban areas with higher dentists’ densities may suffer from supply-side-induced demand [20–22]. Policy-makers are called to action to explore and counter-tackle these differences in servicing, for example by controlling workforce flows or setting incentives for service provision in rural areas.

This study has a number of strengths and limitations. First, it is one of few longitudinal investigations in the very old and comprises a cohort of over 400.000 individuals from three different federal states. Second, a wide variety in possible factors associated with periodontal utilization were assessed, some of them rarely investigated in dental services utilization studies. Third, and as a limitation discussed above, claims data suffer from a range of biases. For example, we cannot easily infer from claimed to provided or even needed treatments. Other relevant factors (e.g., medication, care status) were not available and accounted for, and some available factors (e.g., social hardship status, place of living) came with very limited granularity. Fourth, as utilization was so low, the overall numbers of individuals who claimed certain services are, considering the usual size of routine data analyses, rather small. Notably, with more than 6000 followed individuals receiving periodontal treatments and > 100,000 having been measured using the PSI, our sample still allows for statistically robust analyses. Nonetheless, especially the fit of our regression model was extremely low, indicating that other aspects beyond the covariates assessed determine utilization, but also reflecting the difficulty of an extremely imbalanced dataset. Fifth, individuals insured by AOK Nordost are not fully representative for all Germans, as they are less affluent and tend to be older than the national average. The rural-urban disparities are also more severe in this region, with Berlin as capital and some of the poorest and most rural German municipalities being spatial neighbors. Moreover, privately insured individuals are not at all reflected; these are usually more affluent, better educated, show a more regular utilization pattern and better dental health. Notably, their share is very low in this part of Germany (mainly as entry into the private insurance is by-large income-dependent). Last, an item which is also part of the periodontal treatment chain (occlusal adjustment, BEMA 108) was not separately assessed by our study, mainly as it can be claimed during other treatments, too. Also, and as discussed, we did not assess surgical care in further detail, by large because it was irrelevant in this cohort.

In conclusion, and within the limitations of this study, the utilization of periodontal services in the very old in Northeast Germany was low, and even screening was only performed in a minority of individuals. Policies to increase identification and management of periodontitis especially in the most vulnerable individuals, which are the very old, the sick, the poor, and the rural living ones, are needed.

## Electronic supplementary material

ESM 1 (DOCX 19.2 kb)

## Data Availability

Data used in this study cannot be made available by the authors given data protection rules but may be requested at the GEWiNO.
